# How the inner repetition of a desired perception changes actual tactile perception

**DOI:** 10.1038/s41598-024-53449-7

**Published:** 2024-02-06

**Authors:** Kasia A. Myga, Esther Kuehn, Elena Azañón

**Affiliations:** 1 Leibniz Institute for Neurobiology, Brenneckestrasse 6, 39120 Magdeburg, Germany; 2 Medical Faculty, Otto-Von-Guericke University, Magdeburg, Germany; 3 Hertie Institute for Clinical Brain Research (HIH), Tübingen, Germany; 4 German Center for Neurodegenerative Diseases (DZNE), 72076 Tübingen, Germany; 5 Institute for Cognitive Neurology and Dementia Research (IKND), Otto-Von-Guericke University, Magdeburg, Germany; 6 Center for Behavioral Brain Sciences, Magdeburg, Germany; 7 Center for Intervention and Research on Adaptive and Maladaptive Brain Circuits Underlying Mental Health (C-I-R-C), Jena-Magdeburg-Halle, Germany

**Keywords:** Neuroscience, Physiology, Psychology

## Abstract

Autosuggestion is a cognitive process where the inner repetition of a thought actively influences one’s own perceptual state. In spite of its potential benefits for medical interventions, this technique has gained little scientific attention so far. Here, we took advantage of the known link between intensity and frequency perception in touch (‘Békésy effect’). In three separate experiments, participants were asked to modulate the perceived intensity of vibrotactile stimuli at the fingertip through the inner reiteration of the thought that this perception feels very strong (Experiment 1, n = 19) or very weak (Experiments 2, n = 38, and 3, n = 20), while they were asked to report the perceived frequency. We show that the task to change the perceived intensity of a tactile stimulus via the inner reiteration of a thought modulates tactile frequency perception. This constitutes the first experimental demonstration that an experimental design that triggers autosuggestion alters participants’ tactile perception using a response orthogonal to the suggested variable. We discuss whether this cognitive process could be used to influence the perception of pain in a clinical context.

## Introduction

The idea that ‘thoughts create your reality’ is popular in modern life, because the consequence is that an individual can significantly impact on its own life circumstances, emotional well-being, and health outcomes. The concept of autosuggestion originates from the pioneering work of Coué in the early twentieth century who observed that people’s mental states influence the outcome of medical interventions^1^. Autosuggestion posits that individuals can influence their own mental and physiological states through the repetition of a thought, a so-called suggestion^1–3^. A popular example of autosuggestion is positive affirmations people make to themselves to boost their experiences^2^.

Despite its potential benefits in enhancing everyday life experiences, autosuggestion gained little scientific attention, while different forms of heterosuggestion (i.e., suggestions coming from another person), such as hypnosis or placebo, are more widely studied. Heterosuggestion has been shown to reduce anxiety^4,5^, to support stress management^6,7^, to improve wellbeing^8,9^, and to modulate cognitive performance^10,11^ as well as to increase the perceived control of one’s own actions^12^. Studies on heterosuggestion often focus on health outcomes such as alleviating pain^13,14^. Importantly, these changes are accompanied by altered neurophysiological brain activity (for a review see^15^). Only a limited number of studies show the potential beneficial effects of autosuggestion on personal wellbeing, such as an improvement in quality of life^16^, or changing attitudes towards food^17^. Initial evidence shows that implementing autosuggestion during meditation also alters the activation of different brain networks, including prefrontal and insular cortices^17,18^.

Yet, it appears that most existing studies lack a consistent theoretical and methodological approach to examining the phenomenon of autosuggestion. For instance, different phenomena are intermixed, such as autosuggestion with autogenic training, or imagery^18^. Another common drawback of autosuggestion research is the use of explicit measurements^17^, where results are influenced by demand characteristics^19^. For example, participants’ responses might be biased, leaning towards what they believe the study aims to prove^20^. To overcome the above-mentioned limitations, we provide here a first attempt to test the efficacy of the inner reiteration of a thought in modulating perception while reducing the influence of demand characteristics. To this aim, we have developed an experimental paradigm that relies on the use of implicit measurements to alter an individual’s tactile perception.

In the present study, we define autosuggestion (or self-suggestion) as the rehearsal (i.e., reiteration) of particular thoughts or statements (e.g., ‘the touch I receive is very strong’), with the aim of actively influencing one’s own perception^2^. Even though other definitions of autosuggestion exist^21,22^, this definition is in line with the initial description of autosuggestion proposed by Coué^1^. According to Coué, suggestions are repeated internally by the person using inner speech and/or acoustic-verbal imagery (i.e., speaking in their head). We note that even though we provided this instruction to participants, we cannot be sure which exact cognitive process they employed during the experiment.

We here tested whether autosuggestion can alter participants’ somatosensory perception at the finger. Specifically, we asked participants to reiterate thoughts affirming that the perceived intensity of a given vibrotactile stimulus feels weak or strong. However, they were then tested on the perceived frequency of the touches. In this respect, we made use of the known interaction between vibrotactile intensity and frequency perception, i.e., the ‘Békésy effect’^23^; see also^24,25^. Notably, an increase in the intensity of vibrotactile stimuli, keeping the frequency constant, can lead either to an increase or a decrease in its perceived frequency. Whereas the direction of this effect is different between people, it is usually constant within one individual and can therefore be used to test for the effect of autosuggestion in a within-subject design. We refer to these two effects as ‘positive association’ (an increase in the intensity of a vibrotactile stimulus is accompanied by an increase in its perceived frequency) or ‘negative association’ (an increase in the intensity of a vibrotactile stimulus is accompanied by a decrease in its perceived frequency).

During the experiment, participants were presented with two tactile vibratory stimuli in sequence. While being asked to modulate the perceived intensity of one of the two touches, they were subsequently asked which of the two touches had the higher frequency. Using a similar sequential paradigm, Morley and Rowe^24^ reported that most participants in their sample showed a positive association. We therefore initially targeted participants showing a positive association (Experiment 1). Note that we did not have any specific preferences for either group, as we anticipated obtaining comparable results. We conducted altogether three experiments: Experiments 1 and 2 introduce a new paradigm to investigate the effectiveness of autosuggestion on either increasing (Experiment 1) or decreasing (Experiment 2) the perceived intensity of tactile stimuli indirectly tested via the perceived frequency of these stimuli. The third experiment investigates a possible influence of language on the autosuggestion effect. In Experiment 3, we therefore changed the answer options from ‘low’ and ‘high’ into ‘slow’ and ‘fast’ to circumvent a cognitive association between ‘high amplitude’ and ‘high frequency’ (and vice versa). Together, these three experiments provide a first, systematic evaluation of how autosuggestion influences the perception of touch on the fingers.

## Methods

### Experimental paradigm

In Experiment 1, participants were asked to reiterate the thought that the touch delivered to their left index finger felt very strong, whereas in Experiment 2, they were asked to reiterate the thought that the touch delivered to their left index finger felt very weak. In both experiments, they were asked if the frequency on the right index finger was higher or lower than the frequency on the left index finger.

More precisely, the experimental session consisted of four parts. First, participants were trained to successfully distinguish between tactile intensity and tactile frequency via the presentation of different touches where they had to discriminate the frequency of stimuli (high versus low) while the amplitude of the stimuli raised on each trial. Second, participants who passed the training performed a tactile frequency discrimination task, similar to the main experimental task. This was followed by a training on autosuggestion. Finally, the main experimental session started. The whole procedure, which is detailed below, took 2.5 to 3 h.

In the two conditions that formed the main experimental session (baseline and autosuggestion), participants received two touches in sequence, first on their left index finger (reference finger) and then on their right index finger (comparison finger). The task was to indicate if the touch on the comparison finger was higher/lower in frequency (Experiments 1, 2) or faster/slower in pace (Experiment 3) than the touch on the reference finger. The condition that we refer to as the ‘autosuggestion condition’ was based on the definition of autosuggestion introduced by Myga et al.^2^, and in accordance with the initial description of autosuggestion proposed by Coué^1^. Here, participants were asked to perceive the touches on the reference finger as very strong (Experiment 1) or as very weak (Experiments 2, 3) by internally repeating the thought ‘The touch feels very strong’ or ‘The touch feels very weak’, respectively. It was unknown to participants that in fact the frequency of touches on both fingers remained the same throughout the task. Any alterations in the perceived frequency were therefore due to our experimental manipulation. The intensity on the reference finger was also constant. Only the intensity on the comparison finger varied between experimental trials, however, this was the same for the baseline and autosuggestion conditions. Framed in another way, we asked participants to modulate the perception of the intensity of touch on their left, reference finger but asked them to report frequency perception judgments, rather than perceived intensity, on their right, comparison finger as compared to the left. Given the frequency was in fact the same in each trial, and alterations in the intensity were the same between experimental conditions, we could here test how the inner reiteration of a thought that is supposed to influence intensity perception influences perceived frequency.

## Experiment 1

In Experiment 1, we investigated whether autosuggestion *increases* the perceived intensity of touch on the reference finger. We asked participants to ‘Feel the touch on the left finger as strongly as possible’, using autosuggestion. They were then asked to decide whether the frequency on the comparison finger was higher or lower than the frequency on the reference finger. It was unknown to participants that the frequency was in fact held constant on both fingers (always 30 Hz), also the intensity on the reference finger was always the same.

### Participants

To date, no previous study exists on the effect of autosuggestion on tactile perception based on which we could reliably calculate a desired sample size. We thus based our sample size calculation on the results provided by Fardo et al.^26^. They used imagery as an experimental manipulation to increase or decrease painful sensations. We calculated our sample size based on the effect size of participants’ subjective responses to the intensity of painful stimuli on a visual analog scale, while imaging a bloody wound on the stimulation location (facilitation condition), or without any imagery (baseline condition). Based on the results depicted in Fig. 2a of this empirical study, we calculated the mean and SD for the facilitation condition (mean = 7.45, and SD = 1.60) and the mean of the baseline condition (mean = 6.60). Using the Matlab ‘sampsizepwr’ function for a repeated measures design, with a power set to 0.80, we obtained a required sample size of 30 participants.

Based on this number, we tested N = 33 participants. N = 1 participant reported problems understanding the task during the practice session and did not start the experiment. N = 1 participant did not complete the experiment due to fatigue, and the dataset was destroyed. Out of the 31 remaining participants, n = 10 participants did not meet the goodness-of-fit threshold (R^2^ ≤ 0.40) in one or both conditions. Note that for most participants, a low goodness-of-fit was driven by a lack of a perceptual intensity-frequency coupling (i.e., neither positive nor negative association, ‘flat trend’). N = 2 participants showed a negative association (with negative just noticeable difference mean values on both conditions). Given the low number of participants with a negative association (n = 2) and given that it was not our initial sample of interest, these were removed from analysis. N = 19 participants were analyzed all of whom showed a positive association (8 females, mean age = 28.42, SD = 3.20, 18 right-handed, mean = 92.94, min = 11.11, max = 100; as assessed by Edinburgh Hand Inventory^27^). All participants gave written informed consent and were paid for their participation. The procedures were approved by the Ethics Committee of the Otto-von-Guericke University Magdeburg (ethics code 01/19). All methods were performed in accordance with the guidelines and regulations set out by the ethics committee of the Otto-von-Guericke University Magdeburg and in compliance with guidelines defined by the Leibniz Institute for Neurobiology (LIN) in Magdeburg, where testing took place.

### Procedure

The day before participating in the experiment, participants received a short document with an introduction to autosuggestion, and an outline of the task (this document as well as all available data and scripts can be accessed here: https://osf.io/7hd5a/?view_only=321a7ad916e64f53a11596eccd55bd7e). There were two reasons why these documents were sent to participants: First, we wanted participants to get familiarised with the phenomenon of autosuggestion beforehand. Second, we intended to address motivated participants to take part in the experiment. Note that willingness/intention to create specific perceptual states is assumed to be a precondition to successfully performing autosuggestion (see^2^). In case participants did not like the experimental paradigm, they were offered the option to resign before the experiment took place.

On the testing day, before starting the experiment, participants filled out a consent form as well as the Multidimensional Iowa Suggestibility Scale using a 5-point Likert scale (MISS^28^). We used the Sensation Contagion (SC), Physiological Reactivity (PHR), Psychosomatic Control (PSC) subscales, as well as an overall score of these subscales. The use of these suggestibility scales aimed at revealing participants’ suggestibility regarding concepts that are related to somatosensation and the body.

### Materials

Participants were seated in front of a monitor (24.4-inch screen), with an eye distance of about 56 cm away from the screen. After adopting the most comfortable position, tactile stimuli solenoids (Solenoid Tappers, MSTC3-10M, M & E Solve) were attached to their right and left distal pads of index fingers. Square wave stimuli of 500 ms duration were delivered, one per fingertip, producing a vibrating tapping sensation. Stimuli were presented using MATLAB version R2015a (MathWorks) and Psychtoolbox, version 3.0.11^28^. Each of the four experimental tasks was explained to participants before starting the session. Participants listened to white noise during every task to mask the auditory noise produced by the tappers. Additionally, a sham skin conductance device was attached to participants’ left index finger in the main experimental part (see below).

### Experimental conditions

#### Frequency-intensity discrimination training session

The frequency-intensity discrimination training aimed to familiarize participants with the task and to clarify the difference between tactile frequency and tactile intensity. The experimenter first demonstrated on the dorsum of her own left hand, how vibrotactile intensity and frequency were defined in the present experiment. Specifically, the experimenter applied touches on her own left hand with her right index finger. These touches imitated the up and down movements of the vibrating tip of the stimulus in an exaggerated way. That is, to represent the pressure strength, the experiment repeatedly poked her own hand with larger or smaller up and down movements. In a similar way, to illustrate the dimension of frequency, the experimenter applied multiple pokes on her own hand (approximately 5 in a row) presented one after another with varying speeds. Participants confirmed understanding the difference between vibrotactile intensity and frequency qualities before proceeding to the task.

The practice session consisted of two blocks containing eight trials each. In each block, from trial to trial, the intensity of the stimulation rose step by step, equally on both fingers from level 2 to level 9 in that order (of a 16 V amplifier with 10 as a maximum value). The frequency of each pair was randomly selected in pairs: 10–30 Hz, 30–10 Hz, 20–40 Hz, and 40–20 Hz. The first value corresponded to the frequency delivered to the reference finger, whereas the second value corresponded to the frequency delivered to the comparison finger. Each stimulus lasted 500 ms and the two stimuli were separated by a 1 s interstimulus interval (ISI). Participants were asked to indicate whether the touch on the comparison finger (right finger) was higher or lower in frequency than the touch on the reference finger (left finger). In that way, participants were trained to pay attention to frequency while ignoring the intensity. Responses were given by lifting the toes of the right foot for ‘higher’ or the heel for ‘lower’. An error message was displayed for 2 s on the screen when the foot was lifted outside of the response window. Responses were not timed, but participants were advised to respond as quickly and accurately as they could. After each response, written feedback: ‘Correct’ or ‘Wrong’ appeared on the screen. Participants were allowed to repeat the practice session until they could perform the task successfully. Most participants produced 5 errors or fewer (out of 16) in the last practice session, with the following group averages: Exp 1 = 2.25 errors; Exp 2 positive trend = 2.75 errors; Exp 2 negative trend = 1.22 errors; Exp 3 = 1.6 errors. Three participants (n = 1 in Exp 1, and n = 3 in Exp 3) showed 6 to 7 errors, and n = 1 mistaken the response mapping in Experiment 1 (12 errors). They were permitted to proceed with the experiment as they demonstrated an understanding of the task through verbal communication, despite encountering challenges in discerning the 20 Hz threshold differences used in this context. Note that exclusion of these participants (2 in Exp 1 and 3 in Exp 2) gives a similar pattern of results in Experiments 1 and 2.

#### Training session of the main experiment

After the frequency-intensity discrimination training, participants were trained on the main paradigm. This was similar to the frequency-intensity discrimination training, with the difference that here, touches applied to the reference finger always had the same intensity (level 5 where 10 is the maximum, of a 16 V amplifier). Intensity on the comparison finger varied between 2 and 8 units, in 7 levels. The frequency of stimulation was kept constant on both fingers at 30 Hz (16.67 ms pin up and 16.67 pin down) for a 500 ms stimulus duration. The choice of the used frequency was based on the study by Morley and Rowe^24^. There was no feedback provided. Participants performed 70 trials and had one untimed break in between.

#### Autosuggestion training

Next, participants were trained to perform autosuggestion. They were asked to read again the information sheet they received the day before. The information sheet contained the definition of autosuggestion, the relevance of the concept for the experiment, and an explanation on how to perform autosuggestion (document can be accessed here: https://osf.io/7hd5a/?view_only=321a7ad916e64f53a11596eccd55bd7e). Then, the experimenter removed the tactile stimulator from the comparison finger, and participants only received stimulation to their reference finger. At the beginning of the training, participants received five touches (30 Hz, 500 ms, level 5) separated by a 1 s ISI. These touches were supposed to familiarize participants with the type of stimulation they would receive throughout this training session (always the same). There were 10 trials thereafter. On each trial, participants first received three touches that they were asked to attend without any cognitive modulation. Next, a fixation cross appeared on the screen for 5 s. Participants were instructed to autosuggest during this time that the next three touches would feel as strongly as they possibly could. After each trial, participants marked on a visual analog sliding scale how much they believed they managed to feel the touch as stronger than the non-autosuggested touches. To be able to make this internal and subjective comparison, the stimulation was always the same and participants were informed about that.

### Main experimental session (testing the effects of autosuggestion versus baseline)

Participants placed their hands palm upwards on a table while being seated, with hands about 15 cm apart from one another. Participants underwent two conditions: autosuggestion and baseline, in an ABBA design (first condition counterbalanced across participants). Only the instructions were displayed on the screen. During tactile stimulus presentation, the screen was black. This was done to ensure that participants’ attention was not distracted by any visual marks presented on the screen, such as fixation cross. Before the experiment started, participants indicated on a visual analog slide scale their belief in their ability to be able to change the perception of touches on the reference finger as very strong. This was an expectancy measure, with values between 0 on the left end—not at all, to 100 on the right end- very convinced. In the autosuggestion blocks, the question about their belief that their autosuggestion worked (self-efficacy) was further asked every 14 trials (7 times altogether). This was done to increase the attention towards the perceived effects of their autosuggesting efforts.

**Figure 1:**
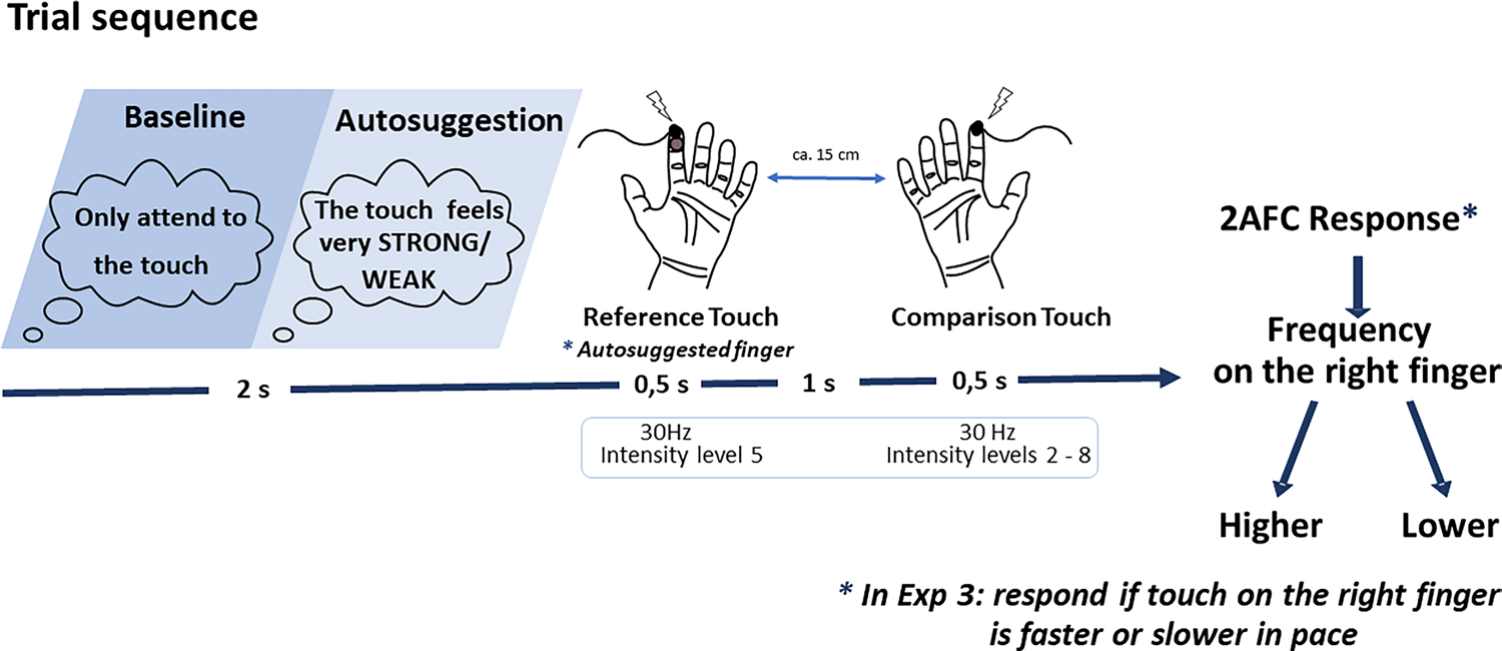
Overview of the main experimental session. Participants placed their hands palms upwards, with hands about 15 cm apart from one another. In both baseline and autosuggestion conditions, participants received two touches: first on the distal pad of their left index finger (reference) followed by the touch on the distal pad of their right index finger (comparison). After the second touch was delivered, participants were asked to indicate whether the touch on the comparison finger was higher or lower in frequency (Exp 1 and Exp 2) or faster or slower in pace (Exp 3) than the touch on the left reference finger, while ignoring the intensity. Before the touch delivery (during the intertrial interval, ITI of 2 s), in the autosuggestion condition, participants autosuggested that touch on the left, reference finger felt as strong (Exp 1) or as weak (Exp 2 and 3) as the participant could possibly modulate it. In the baseline, participants were instructed only to attend to both incoming touches. The frequency on both fingers was always the same (30 Hz) and only intensities on the right index finger varied across 7 intensities. The intensity on the left finger was stable and set out to the intensity of the middle range of those on the right finger. An additional sham device to monitor skin conductance was attached to the reference finger in the main experimental part, to motivate participants (see “Main experimental session testing the effects of autosuggestion versus baseline” section).

In the autosuggestion condition, participants were first asked to create thoughts of perceiving the upcoming touches on the reference finger as very strong for one minute and then every 15th trial for another period of 10 s (7 times in total). To reach this goal, they were instructed to internally repeat the desired outcome. Participants were instructed to avoid creating thoughts using negations in the wording (e.g., ‘the touch is not weak’). We also asked them to avoid creating images in their mind’s eye as much as possible. This aimed to reduce the likelihood to use visual imagery as a strategy to solve the task. The trials were separated by 2 s intertrial intervals (ITIs), during which participants were asked to repeat their suggestions. On every trial, participants received a tactile stimulus (500 ms) to the reference finger, followed, after a 1 s ISI, by a tactile stimulus (500 ms) to the comparison finger, always in that order (see Fig. 1). They then indicated via a foot press if the touch received on the comparison finger was higher or lower in frequency than the touch received on the reference finger (while ignoring the intensity). It was unknown to participants that the frequency of the stimulation in both fingers was in fact always the same (30 Hz). The intensity on the reference finger was also constant (level 5). Only the intensities on the comparison fingers varied from 2 to 8 (10 is maximum) of a 16 V amplifier, in 7 levels. Participants were allowed one untimed break in each block. During the whole experiment, a sham skin conductance device was attached to their reference finger. This was done to leave participants under the impression that their level of autosuggestion could be measured by us. Participants were told that based on the physiological measurements collected by this device, the experimenter could calculate if their autosuggestion was successful or not, and to which extent. In fact, this was not the case, however.

The baseline condition was similar to the autosuggestion condition. However, here, instead of internally repeating thoughts, participants were instructed to simply attend to the touches. Note that in both autosuggestion and baseline conditions, participants were instructed to focus their attention equally to each touch, given that the task required a comparison of sensations across the two touches. More precisely, participants were instructed to first attend to the reference (autosuggested) touch, and subsequently to the comparison touch. After half of the experiment was done (i.e., before the start of the third block), the experimenter swapped the tactile stimuli solenoids between the two fingers to balance across conditions any possible differences in the strength or qualia of the stimuli applied to each hand driven by potential physical differences between the two solenoids.

After finalising all four blocks that composed the main experiment, participants indicated on an analog slide scale their belief in how much they felt the touch on their left finger stronger when autosuggesting so (i.e., final self-efficacy measure). We delayed immediate disclosure about the fact that the skin conductance device was in fact not measuring their performance success, due to concurrent studies that utilised a similar autosuggestion procedure, combined with placebo. Our decision aimed to minimise the risk that participants in these parallel studies question the credibility of the main experimenter. Consequently, we chose to defer this disclosure until after all data collection was completed.

### Analyses

Analyses were performed in MATLAB version R2015a (MathWorks). Participants’ responses were extracted as a proportion of responses where the comparison stimuli were judged as higher and fitted as a function of the intensity of the comparison stimuli using a logistic function. An unbiased participant should produce a flat line (all responses about 50%), when asked to compare the frequency across the two hands, given that in fact, the frequency was always constant across the entire experiment. However, due to the above-mentioned associations in perception between intensity and frequency, we expected in most participants the logistic functions to produce a rising (‘positive association’) or decreasing (‘negative association’) fit. To decide whether a participant produced a rising or decreasing psychometric function, we calculated the just-noticeable difference (JND) as the semi-interquartile range. Note, that here, the JND cannot be considered as a measure of precision, given that larger or smaller JNDs indicate the strength of the ‘perceptual illusion’ between intensity and frequency, rather than how precise participants discriminate between stimuli. A positive JND value means that the psychometric function is rising (i.e., the participant showed a ‘positive association’). A negative JND value indicates that the psychometric function is either decreasing (i.e., the participant showed a ‘negative association’) or the entire psychometric curve is shifted upwards but slightly rising. We therefore used both visual inspection of the curve’s trend and JND values to decide whether a participant produced a rising or decreasing psychometric function. In addition, only data that met a goodness-of-fit criterion (R^2^ ≥ 0.40) were included in the final analyses. The Point of subjective equality (PSE) was extracted as our main dependent variable, which here corresponds to the intensity of the comparison stimuli at which the two stimuli are considered by the subject as having the same frequency. Morley and Rowe^24^ observed a larger sample of participants (5 out of 8) with a positive association trend. Thus, only participants showing a positive association (positive JNDs) were considered in Experiment 1.

Due to the sample size being smaller than required to reach a predefined effect size, to determine if differences across conditions were statistically significant, we used the non-parametric Wilcoxon Signed-Ranks test^29^ using the Exact Tests™ software. The Wilcoxon Signed-Ranks test is the non-parametric equivalent of the paired-sample t-test^30^. The Exact Tests™ is a statistical package built into the Statistical Package for the Social Sciences software (SPSS). This method allows making reliable inferences in cases when data is not normally distributed, sparse, heavily tied, or when datasets are small^29^. The algorithms used by the software compute exact p-values for hypotheses testing, based on the exact distribution of the test statistic. Therefore, these tests do not rely on assumptions that data confirms to a particular distribution. The significance level to determine a significant effect was alpha < 0.05. The corresponding Wilcoxon effect sizes (r) were calculated using the following equation:

$$\mathrm{r }=\mathrm{ Z}/\sqrt{N,}$$

where Z is the z-score, and N is the total number of observations over the two conditions^31^. The expectancy ratings and final self-efficacy beliefs were compared using non-parametric Wilcoxon Signed-Rank Tests in Jeffrey’s Amazing Statistics Program (JASP), version 0.17.1.0. The significance level to determine a significant effect was alpha < 0.05. For each of the three suggestibility subscales tested (MISS^31^), item ratings were summed up. The overall suggestibility score was calculated as the sum of all subscales. Scores ranged between 12 and 60 for the Sensation Contagion scale, 13–65 for the Physiological Reactivity scale, 15–75 for the Psychosomatic Control scale, and 40–200 for the overall suggestibility score. A higher score indicates a greater level of suggestibility.

### Results experiment 1

#### PSEs

**Figure 2:**
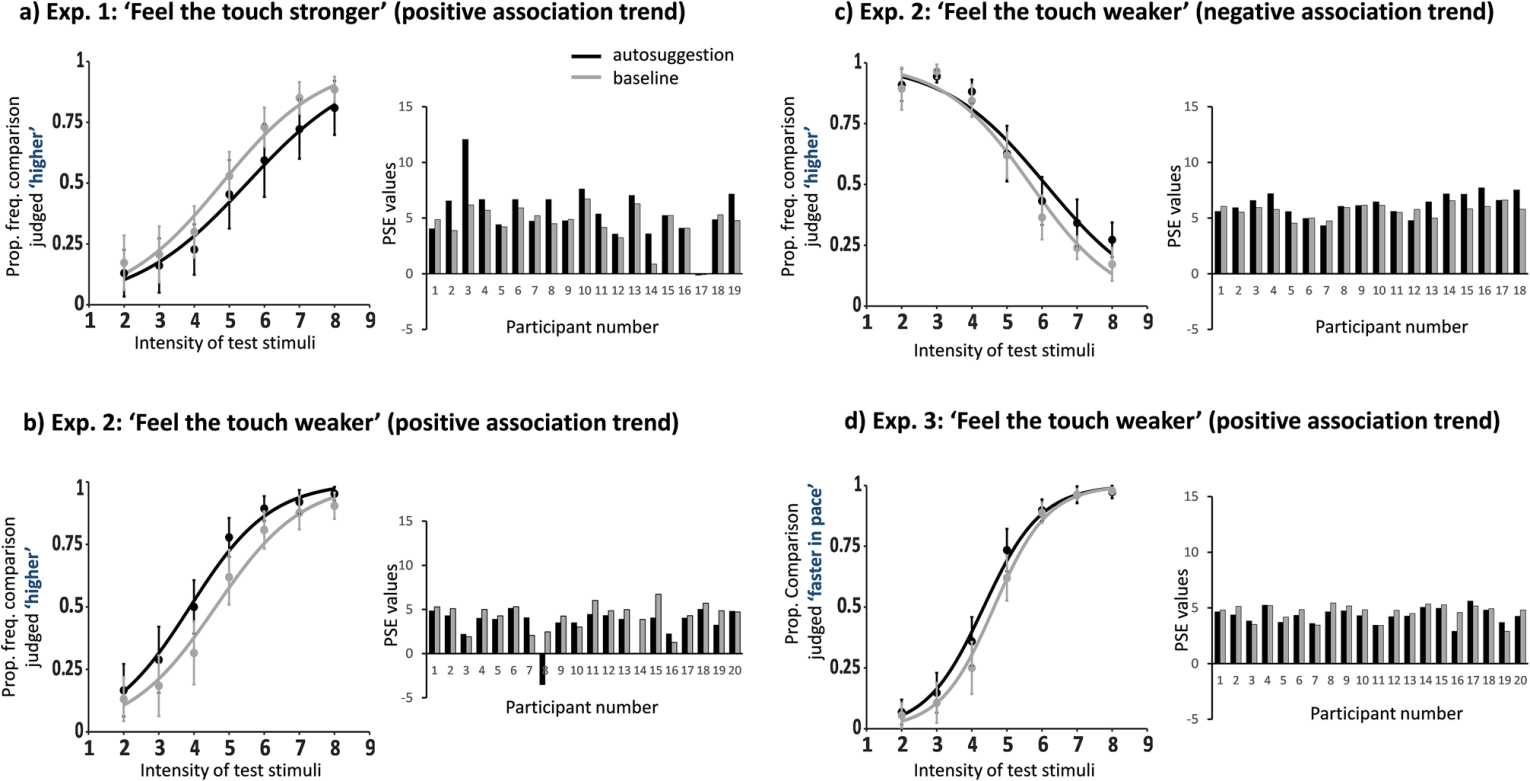
Results of all experiments. The black color depicts autosuggestion results and the grey color—results in the baseline. The Y-axis of the psychometric graphs in Experiments 1 (**a**) and 2 (**b,c**) represent the proportion of responses where the comparison finger was judged as higher in frequency than the frequency at the reference finger. For Experiment 3 (**d**), the Y axis represents the proportion of responses where frequency at the comparison finger was judged as faster in pace than frequency at the reference finger. Note, that frequency on both fingers was always the same: 30 Hz. The X-axis of the psychometric graphs represents the levels of stimuli intensities applied to the comparison finger, from level 2 to level 8. Note that intensity on the reference finger was always set to level 5. (**a**) In participants with a positive association trend after autosuggesting that the touch on the reference finger felt very strong, participants’ frequency perception was significantly higher at the reference finger in the autosuggestion condition compared to baseline. (**b**) This effect was reversed when a new sample was asked to autosuggest that the reference finger felt very weak. (**c**) In participants with a negative association trend after autosuggesting that the touch on the reference finger felt very weak, participants’ frequency perception was significantly lower at the reference finger in the autosuggestion condition compared to baseline. (**d**) A new sample of participants with the positive association trend was tested (Experiment 3), autosuggesting a weaker feeling of touch applied on the reference finger but judging the speed of pace perception. These results parallel the results obtained by participants with the positive association trend in Experiment 2 (**b**). Error bars represent 95% CIs. Bar graphs represent individual mean PSE values from participants obtained in the autosuggestion and baseline conditions. Visual inspection of (**b**) might suggest that participant number 8 is an outlier. This participant, however, passed through all exclusion criteria. Yet, further analysis without this participant produces a similar pattern of results (Z =  − 2.173, p = 0.029, r = 0.50).

Given in Experiment 1, we included only people showing a positive association, if autosuggestion was successful in producing the perception of a stronger percept at the reference finger, frequency perception should increase in that finger. In other words, the greater the perceptual intensity experienced on the reference finger, the higher the corresponding perceptual frequency should be. Conversely, the perceptual intensity experienced on the comparison finger should be lower as compared to the reference finger. As such, the corresponding perceptual frequency should also be perceived as lower. Therefore, in the autosuggestion condition, participants should report more often that the comparison stimuli had a lower frequency compared to the baseline condition. This should produce a bias towards larger PSE values in the autosuggestion condition compared to the baseline condition, and the psychometric curve should shift towards the right side of the baseline curve. This pattern was confirmed given the PSE in the autosuggestion condition (mean = 5.52, SD = 2.40) was indeed significantly higher compared to the baseline condition (mean = 4.52, SD = 1.71), Z =  − 2.415, p = 0.014, r = 0.39 (see Fig. 2a).

#### Expectancy and suggestibility scores

Participants’ mean expectancy score for succeeding in autosuggestion prior to starting the task was 55.18 (SD = 24.51, where 0 indicates minimal belief and 100 indicates maximal belief). The mean self-efficacy rating after finishing the task on the same scale was 47.15 (SD = 28.76). These ratings did not differ significantly, Z = 1.569, p = 0.123, r = 0.41.

On average, participants scored m = 23.21 (SD = 6.43) on the Sensation Contagion scale, m = 46.32 (SD = 8.59) on the Physiological Reactivity scale, m = 38.74 (SD = 9.18) on the Psychosomatic Control scale, and m = 108.21 (SD = 17.28) as an overall score of suggestibility characteristics. Participants scored around average (based on norms acquired by Kotov et al. 2004^31^). There were no outliers, as calculated by 3 SDs above the norms.

## Experiment 2

In Experiment 2, we investigated whether autosuggestion is effective in *decreasing* the perceived intensity of touch applied on the left index finger. Consequently, in Experiment 1, we asked participants to ‘feel the touch on the left finger as weak as possible’, using autosuggestion, and again to judge whether the frequency on the right finger was higher or lower than the frequency on the left.

### Participants

Similar to Experiment 1, our group of interest were those with a positive association. We therefore intended to stop sample collection once we had acquired 20 datasets that exhibited a positive association trend and met the same inclusion criteria used in Experiment 1. To reach this goal, N = 54 participants were tested. Datasets of n = 15 participants were removed from the analysis due to poor goodness-of-fit threshold (R^2^ ≤ 40). One participant withdrew consent from the study, and the data were destroyed. N = 20 formed our sample of interest (9 females, mean age = 26.80, SD = 3.04). Unexpectedly, n = 18 participants showed a negative association (note that there were only n = 2 during the testing of Experiment 1). Given the large number of participants with a negative association tested in Experiment 2, these were this time included as a second, independent group into the analyses post hoc (8 females, mean age = 26.44, SD = 4.46). Most participants were right-handed except for one left-handed and one ambidextrous participant (mean = 84.67, min = -89.47, max = 100; as assessed by Edinburg Hand Inventory^27^). All participants gave written informed consent and were paid for their participation. The procedures were approved by the Ethics Committee at Otto-von-Guericke University Magdeburg (ethics code 01/19). All methods were performed in accordance with the guidelines and regulations set out by the Ethics Committee at Otto-von-Guericke University Magdeburg and in compliance with guidelines defined by the LIN, where testing took place.

### Procedures

The experimental procedure was the same as in Experiment 1, except that, in Experiment 2, participants were asked to change the perceived intensity on their left finger towards a perceptually *weaker* intensity. They were thus guided to create thoughts such as ‘the touch on the left feels very weak’. In this experiment, we administered an additional questionnaire: The Spontaneous Use of Imagery Scale (SUIS^32^), to explore the level of spontaneous imagery use in daily life.

### Analysis

Analyses were identical to those in Experiment 1. SUIS scores were added up to indicate the level of participants’ spontaneous imagery use. The score ranges between 12 and 60, with higher scores indicating greater involvement of imagery. Additionally, we used a two-sample non-parametric Mann–Whitney U-test in JASP, version 0.17.1.0 to compare the mean expectancy and self-efficacy scores between the group with the positive association and the group with the negative association. The level to determine a significant effect was alpha < 0.05. The effects sizes were given by the rank biserial correlation.

### Results experiment 2

#### PSEs

We now analysed two groups, the positive association group and the negative association group. If autosuggestion was successful in producing the perception of *weaker* intensity at the left (autosuggested) finger in the positive association group, then frequency perception should decrease in that finger. Therefore, in the autosuggestion condition, participants should report more often that the comparison stimuli had a *higher* frequency compared to the baseline. This should produce a bias towards *smaller* PSE values in the autosuggestion condition compared to the baseline condition, and the psychometric curve should shift towards the *left* side of the baseline curve.

In the negative association group, the perception of a weaker percept at the left (autosuggested) finger should produce an increase in frequency perception in that finger. Therefore, in the autosuggestion condition, participants should report more often that the comparison stimuli had a lower frequency, as compared to baseline. This should produce a bias towards smaller PSE values in the autosuggestion condition compared to the baseline condition, and given the negative fit, the psychometric curve should also shift towards the left side of the baseline curve.

Figure 2 shows the results for participants with a positive association (2b) and a negative association (2c). As in Experiment 1, datasets of both groups were analysed using the Exact Tests™ for Wilcoxon Signed-Ranks test. In participants with a positive association, as expected, the PSE values in the autosuggestion condition (mean = 3.40, SD = 2.02) were significantly lower as compared to the baseline condition (mean = 4.30, SD = 1.46), Z =  − 2.389, p = 0.015, r = 0.38). During autosuggestion, the frequency perception on the autosuggested finger was lower compared to baseline, which is reflected by participants responding more often that the comparison finger had a higher frequency. Visual inspection of Fig. 2b might suggest that participant number 8 is an outlier. However, this participant passed through all our exclusion criteria. Yet, further analysis without this participant produces a similar pattern of results (Z =  − 2.173, p = 0.029, r = 0.50).

Counterintuitively, in participants with a negative association, the mean PSE was significantly greater in the autosuggestion condition (mean = 6.21, SD = 0.96) compared to the baseline condition (mean = 5.72, SD = 0.58), Z =  − 2.199, p = 0.027, r = 0.37). Participants therefore here responded more often that the comparison finger had a higher frequency than the autosuggested finger. Note that the opposite trend would be expected if autosuggestion indeed reduced the perceived intensity.

#### Expectancy, suggestibility, and imagery scores

The mean expectancy score for succeeding in autosuggestion before starting the task was m = 39.45 (SD = 28.43) for participants with a positive association, and m = 52.00 (SD = 24.65) for participants with a negative association. The mean self-efficacy ratings after finishing the task were m = 39.93 (SD = 27.96) and m = 49.54 (SD = 28.29), respectively. These ratings did not differ significantly before and after the experiment (positive association Z = − 0.149, p = 0.898, r = 0.01; negative association: Z =  − 0.479, p = 0.648, r = 0.02). In addition, there was no significant difference between mean expectancy ratings U = 230.00, p = 0.149, r = 0.278 or mean self-efficacy ratings U = 217.50, p = 0.279, r = 0.208 between participants with positive and negative associations.

On average, participants with a positive association scored for positive and negative trends respectively, m = 22.40 (SD = 5.45) and m = 22.39 (SD = 6.84) on Sensation Contagion scale, m = 46,70 (SD = 10.78) and m = 46.44 (SD = 6.55) on Physiological Reactivity scale, m = 39.55 (SD = 9.58) and m = 38.56 (SD = 11.31) on Psychosomatic Control scale, and m = 108.65 (SD = 22.47) and m = 107.39 (SD = 20.05) as an overall score of suggestibility characteristics. Participants were thus average-suggestible based on norms acquired by Kotov et al.^31^. Participants’ mean score on the Spontaneous Use of Imagery scale was m = 41.45 (SD = 7.69) and m = 38.33 (SD = 7.33) for positive and negative trends, respectively, indicating the average use of spontaneous imagery in daily life (based on norms gathered on a sample size of N = 491 by Nelis et al.^32^). In both groups, there were no outliers, as measured by 3 SDs above the norms.

## Experiment 3

In Experiment 3, we wanted to ensure that the results obtained in the previous two experiments reflected changes in perception and not a general response bias. In the previous experiments, participants were asked to feel the touches ‘stronger’ or ‘weaker’; this could interfere with the task to indicate whether the frequency feels ‘higher’ or ‘lower’, as these words are closely connected. A Google search, for instance, returns a substantial 88 million results for “high intensity” and 36 million results for “low intensity”. Considering the interchangeability of these terms, it is plausible that participants, after repeatedly associating the touch on the reference finger as “weaker” (i.e., lower) in intensity, may have been more inclined to report the comparison finger as “higher” in frequency. To overcome this issue, in Experiment 3, we used words that do not easily associate with ‘stronger’ or ‘weaker’, i.e. ‘faster’ and ‘slower’. It’s worth noting that the terms “fast intensity” and “slow intensity” yield relatively fewer Google entries, with only 44,000 and 17,000 entries, respectively. This suggests that frequently associating the reference finger’s touch as “weaker” in intensity is unlikely to result in participants reporting more frequently the comparison finger to have a “faster pace”. We instructed participants that the pace of the stimuli corresponds to how fast or how slow the stimuli was touching the skin on each tap within the 500 ms vibration, to increase the attention towards the pace. We stressed this by repeatedly tapping with the experimenter’s index finger on the top of the participant’s left hand to demonstrate what fast and slow meant. We asked participants in Experiment 3 to use the technique of autosuggestion to make the perceived intensity feeling weaker (similar to Experiment 2). This time, we did not send the information sheet to participants before testing. They were only presented with it on the day of the experiment. Due to the long duration of testing (approximately 2.5–3 h), in Experiment 3, we used the Short Suggestibility Scale (SSS)which consists of 21 items selected from all subscales of MISS^31^. Additionally, we asked participants to provide a qualitative description of their strategy to solve the task.

### Participants

As in Experiment 2, we aimed at stopping sample collection after obtaining 20 datasets with a positive association trend. The same inclusion and exclusion criteria were used for Experiment 3 as were used for Experiments 1 and 2. To reach this goal, n = 24 participants were tested. Three datasets were removed due to poor goodness-of-fit (as indicated by R^2^ ≥ 0.40). n = 1 participant showed a negative association trend; this dataset was not considered for analyses. N = 20 participants were analysed for Experiment 3, (5 females, mean age 24.80, SD = 2.04), n = 16 participants were right-handed, n = 2 were left-handed, and n = 2 were ambidextrous (mean = 67.15, min =  − 100, max = 100; as assessed by Edinburg Hand Inventory^27^). All participants gave written informed consent and were paid for their participation. The procedures were approved by the Ethics Committee of the Otto-von-Guericke University Magdeburg (ethics code 01/19). All methods were performed in accordance with the guidelines and regulations defined by the Ethics Committee of the Otto-von-Guericke University Magdeburg and in compliance with guidelines defined by the LIN Magdeburg, where testing took place.

### Procedures and analyses

Procedures and analyses in Experiment 3 were identical to those in Experiment 2, except that, here, participants judged whether the touch on the comparison finger was faster or slower in pace than the touch on the reference finger. Participants filled out a Short Suggestibility Scale (SSS), a subscale of the Multidimensional Iowa Suggestibility Scale (MISS^31^, scores ranging between 21 and 105, a higher score indicating higher suggestibility trait). Additionally, participants were asked to give a qualitative description of their strategy to solve the task.

### Results experiment 3

#### PSEs

Note that in Experiment 3, only participants with a positive association were analysed (see Fig. 2d). Results reveal that the psychometric curve during the autosuggestion condition was shifted towards the left compared to the baseline condition (PSE in autosuggestion condition: mean = 4.34, SD = 0.67, PSE in the baseline condition: mean = 4.62, SD = 0.74, Z =  − 2.240, p = 0.024, r = 0.35). Therefore, even with the altered instructions that avoid the association of responses, the results of Experiment 3 replicate the results of Experiment 2. In both Experiment 2 and Experiment 3, participants with a positive association responded more often in the autosuggestion condition compared to the baseline condition that the frequency on the comparison finger was faster (or higher, in Experiment 2) than the frequency on the reference finger.

#### Expectancy scores

Participants’ mean expectation score for succeeding in autosuggestion before starting the task was 47.22 (SD = 29.25). The mean self-efficacy rating after finishing the task was m = 58.24 (SD = 29.79). These beliefs did not differ significantly, but showed a trend in this direction Z =  − 1.867, p = 0.064, r = 0.09.

On average, participants scored m = 50.85 (SD = 13.98) on the Short Suggestibility Scale, and m = 43.25 (SD = 7.45) on the Spontaneous Use of Imagery scale. All participants were average suggestible (based on norms reported by Kotov et al.^31^) and higher than average imagers (based on norms gathered on a sample size of N = 491 by Nelis et al.^32^). There were no outliers as measured by 3 SDs above the norms.

#### Qualitative reports on how participants performed autosuggestion

14/20 participants stated that they predominantly created and reiterated thoughts that the touch on the autosuggested finger felt weaker. From those, n = 2 participants reported that they additionally used imagery. They did not refer in their descriptions to have used visual imagery, rather, they described to have “imagined perceptions of weaker tactile intensities”. 5/20 participants did not provide a precise description of their applied strategies. 1/20 participant reported that he/she had tried to directly influence frequency perception by autosuggesting. More precisely, the participant reported that the touch on the left (autosuggested) finger ‘should vibrate less and the right-hand index finger should vibrate more’. Note that the net effect of autosuggestion observed in this experiment is not driven by the behavior of this participant as the PSEs of the autosuggestion and baseline conditions were very similar in this participant (PSE autosuggestion = 5.25; PSE baseline = 5.21). To access detailed descriptions of how participants used autosuggestion, please visit the following link: https://osf.io/7hd5a/?view_only=321a7ad916e64f53a11596eccd55bd7e.

## Discussion

Our study shows that the inner reiteration of a thought alters participants’ tactile perception using a response orthogonal to the suggested variable. Specifically, participants internally manipulated, via internal thought repetition, tactile intensity perception on the reference finger (to perceive intensities as higher or lower than they actually were). However, they judged tactile frequency. Given participants were naive about the relationship between tactile intensity and frequency perception, this paradigm avoided demand characteristics to influence the results. In addition, even if participants were making intuitive guesses about the best suited responses, they could not predict the direction of the effect, because the relationship between tactile amplitude and frequency perception varies between participants. Our results show that after the inner repetition of the thought that the touch on the reference finger feels very strong, perceptual judgements of frequency on that finger were higher than in the baseline condition (Experiment 1). Similarly, when participants repeated the thought that touches on their reference finger feel weaker, perceptual judgements of frequency on that finger were lower than in the baseline condition (Experiment 2). This was true for participants with a positive association (i.e., perceiving higher frequencies at higher intensities) in both experiments, and shows that the inner reiteration of a thought alters participants’ tactile perception into the expected (autosuggested) direction.

For those participants with a negative association (i.e., perceiving lower frequencies at higher intensities), autosuggestion produced counterintuitive results. The reiteration of the thought to perceive weaker touches (Experiment 2) resulted in a decrease, rather than an increase, in frequency perception. When participants with a negative association (but not those with a positive association) therefore tried to decrease the perceived intensity of touches on the finger, they reported a perceived frequency change that is associated with the stronger perception of touch. These results are likely not due to different expectations about their own successes as both groups did not differ in their mean expectation scores. One possibility for this result is a response bias. More specifically, after internally repeating the sentence ‘the touch to my left finger feels weaker’, participants might have been more prone to respond ‘touch to the right finger feels higher in frequency’ given both assignments are closely connected. To account for this possibility, in Experiment 3, we asked participants to judge whether touches on the comparison finger felt faster or slower than touches on the reference finger. In this way, we made the two relevant features, intensity (strong versus weak) and frequency (fast versus slow), more distinct. The results of Experiment 3 confirmed our previous findings in the positive association group, suggesting that the described effect is not due to response bias or confusion between intensity and frequency.

Previous studies have shown that the perception of magnitudes can be mutually influenced through a ‘generalised magnitude system’, with the idea that different features such as size, time, or number are processed by a common mechanism (e.g.^33–35^). For example, larger objects are perceived as lasting longer, being brighter, or having a larger number of elements^33^. The existence of participants with a negative association, however, suggests that the coupling between frequency and intensity in touch is not supported by this generic representation of magnitudes as is the case for the processing of other information. However, it might still be possible that the effect of autosuggestion in this task reflects a change in this generalized magnitude system. The idea is that when participants are required to select which of two stimuli is greater in magnitude, in this case, frequency, their judgments are influenced by the magnitude of the stimuli on another dimension, in this case, intensity. Therefore, a change in perception towards a lower stimulus might bias perception towards a lower frequency, regardless of the actual coupling between the two features. In other words, the results obtained here could reflect number processing, where ‘less’ in one dimension (i.e., intensity) is associated with ‘less’ in another dimension (i.e., frequency)^35^. This would be consistent with the results obtained in both samples of participants with negative and positive intensity-frequency associations.

Autosuggestion is unique in the sense that a person is actively producing perceptual changes of his/her own choice. However, the idea that somatosensory perception can be changed via top-down modulation is not new. For instance, orienting attention toward one’s own body enhances the detection and discrimination of cutaneous stimuli^36^, induces spontaneous sensations in the skin^37^, increases pain thresholds^38^, and even modulates the temperature of the attended skin^39^. On the contrary, directing attention away from the body reduces perception of tactile stimuli^40^. In these paradigms, however, the participant is usually not asked to produce or change any specific sensation, which is, however, the key idea of autosuggestion. Rather, the behavioral and neurophysiological effects occur without explicit control by the participant. With respect to mental imagery, participants are usually asked to create one specific, pre-defined image vividly in their minds (i.e., a wounded arm^26^), but they are usually not asked to change their sensation at will. Of note is that these perceptual changes are usually accompanied by corresponding neurophysiological changes (e.g.^17,41^). Drawing attention to one’s own hand, for example, alters neural activation levels in sensorimotor networks and associated attentional processing streams^42^. Also observing another person’s hand being touched induces somatosensory ‘mirroring’ responses in the primary somatosensory cortex, as shown using ultra-high resolution 7 Tesla fMRI^43^.

In our experiments, we asked participants to target their perception of stimulus intensity of non-noxious nature. Indeed, it seems more relevant and ecologically valid to target painful experiences causing suffering, rather than just modifying emotionally neutral, tactile perceptions in a healthy population. Research on discriminative touch such as the present study, provides basic insights into processes involved in somatosensory perception. Here, we showed that perceptual ratings of intensity are not a pure reflection of bottom-up processing, but they can be intentionally influenced by top-down control processes as well. A question arises, whether the present findings from discriminative touch can be extrapolated to pain sensations. Discriminative touch is focused on providing detailed tactile information, while pain is a protective mechanism that warns the body of potential harm. Given that both forms of somatosensory information are processed by different, although overlapping, neural systems^44^, it is not straightforward to assume that effects of autosuggestion on discriminative touch would be similar to those for pain. Testing healthy individuals with pain stimuli, and clinical cohorts, on our paradigm could shed some light on the similarities and differences across the two somatosensory modalities. Nonetheless, it is important to note that nociceptive processing can also be modulated by different cognitive factors, such attention or the likelihood of stimulus occurrence, similar to how these factors affect the processing of touch^45^. Consequently, one might or might not expect analogous outcomes for pain during autosuggestion. Moreover, patients may be more motivated to take part and focus attention on the studies than the healthy population, producing thus more reliable results. This motivation may be evoked by a personal interest in finding treatments or solutions for their existing conditions^46^ or by altruistic reasons^47^. Conversely, healthy participants, belonging usually to the student population and motivated mostly by financial compensation^48^, may lack the efforts in producing equally reliable results.

It is noteworthy that we applied stimulation on spatially separate body parts (contrary to Morley and Rowe^24^, which presented sequential stimuli on the same fingertip). This experimental manipulation, however, facilitates cognitive differentiation between autosuggestion, consistently applied to the left index finger, and the comparison, consistently applied to the right index finger. We expected that it would be less demanding for participants to shift attention away from the task of autosuggestion when the two touches were distinctly and visibly separated in space, than when applied on the same fingertip. It is, however, true that switching attention between two stimuli (here to compare the two stimuli) becomes easier as the external distance between the two stimuli is reduced^49^. Nonetheless, if the effects observed in autosuggestion were uniquely mediated by differences in attention distribution to each finger, we would not observe differential effects between the weak and the strong conditions. Here, indeed, in both conditions, participants were instructed to attend to their left finger while being asked to enhance or reduce their tactile sensations; and then switch their attention to their right finger to give their most accurate difference ratings. They were explicitly informed that they would not be able to give their most accurate difference ratings if they only focused on one finger. Instructing participants to adhere to task demands does not guarantee they will do so. Thus, we cannot be sure whether or not participants’ attention was uniformly directed, in sequence, to both fingers. However, if participants attended more to the autosuggested than the comparison finger with the “stronger” instruction and less with the “weaker” instruction, this would have been reflected in flatter psychometric curves overall in the later condition, rather than in changes in PSEs, which was not the case.

Roy and Hollins^25^ suggested that the ratio of recruitment between Pacinian corpuscles (PA) and rapidly adapting (RA) sensory fibers might be the origin of the frequency-intensity associations discussed in this study. The hypothesis is based on the observation that at specific vibration frequencies, the ratio of PC and RA class recruitment varies in response to changes in vibration intensity. For instance, at lower amplitudes, the activation should predominantly involve PC fibers, whereas as amplitudes increase, a growing proportion of recruited fibers is expected to belong to the RA class (Talbot et al.^50^ for evidence in monkey). Under the assumption that RA and PC sensory fibers exhibit distinct vibration sensitivities, differences in the recruitment rate would consequently manifest as variations in frequency perception. However, Roy and Hollins’ study found the pattern of frequency-amplitude to be well-described by a ratio model in only three out of the four participants examined, and indeed, in those who exhibited a negative trend. Similar mixed indicators regarding the plausibility of the ratio model were noted in a prior study conducted by Morley and Rowe^24^. An alternative suggested hypothesis for the frequency-intensity associations^23,24^ regards the use of a temporal patterning of impulse activity (temporal pattern code), in which responses are phased-locked to the vibration of the tactile stimulus^51^. Thus, at high intensities of vibration, neural interactions might inhibit firing during some of the cycles causing the stimulus to be perceived as lower in frequency. This however, would only explain the negative association but not the positive association. In addition, it has been demonstrated that cortical neurons are not likely to skip a cycle as vibration amplitude increases^52^. To our knowledge, no other hypothesis has been formulated regarding the frequency-intensity interaction discussed here. Nevertheless, it is not surprising that the origin of this interaction remains unknown, given that the exact way in which stimulus frequency and amplitude are translated into perception of pitch and intensity, when considered in isolation, are not clear either^53–55^. In addition, the mechanisms that account for the association between frequency and intensity in touch might differ from the neural mechanisms that determine the subject’s perception of pitch and intensity, and other higher-level cognitive factors such as the generalized magnitude system mentioned above, might be involved. When the neural and peripheral mechanisms that underlie tactile frequency-intensity coupling are clarified, it will be possible to develop hypotheses about why a cognitive manipulation differentially affects people with a positive versus negative association, as shown here.

What brain mechanisms are involved in autosuggestion is an open question, and carefully designed neuroimaging experiments could help understand better the neural processes involved in autosuggestion. Lena et al.^56^ concisely summarized the literature describing the role of the semantic aspect of language and verbal suggestions on pain perception. The authors concluded that the use of pain-related vocabulary is associated with altered pain perception, at behavioral and neural levels. Several mechanisms for these effects have been proposed, for example semantic-related priming^57^. Since our participants were instructed to reiterate words describing the intensity of tactile percepts, one might expect a similar brain network to be related to the effect of autosuggestion in the verbal form. However, further studies exploring this possibility, and also comparing the networks that are involved in autosuggestion with those involved in mental imagery, need to be undertaken to better understand the brain mechanisms underlying autosuggestion.

### Limitations of the study

Despite using an innovative approach to explore the effects of autosuggestion, our study has a few drawbacks. The principal limitation of our study is that it could not separate autosuggestion from the possible confounding effects of other top-down mental practices, such as visual imagery and attention. Note that by visual imagery, we refer here to the usage of images such as a glove to produce the sensation of reduced intensity. While we made it clear to participants that our focus was solely on the effects of the inner repetition of a thought, the potential interference of other cognitive processes cannot be excluded. Also attention might have influenced our results. In Experiment 1, participants were asked to direct attention to the autosuggested finger and to try to feel the touches at a stronger intensity. It can therefore not be determined if attentional enhancement or the will to perceive the touches stronger led to the desired effects. However, in Experiment 2 and Experiment 3, participants directed their attention to the autosuggested finger while being asked to feel the touches at a weaker intensity. Given the direction of the effect was modulated in our studies, attentional enhancement cannot explain these results. However, it is possible that to successfully solve the task in Experiment 2 and Experiment 3, participants directed their attention away from their finger, to, for example other parts of their body, or to the surroundings. This could also have resulted in a success to lower the perceived intensity of touches. Therefore, we cannot exclude that attention influences the results of the present study. However, diminished attention to the autosuggested finger should have impacted the comparative ratings between the two fingers, potentially resulting in flatter psychometric curves in the weaker condition, which was not the case.

Different mental strategies implemented by participants could also explain interindividual differences. To account for that, in Experiment 3, we included an open question at the end of the experiment, where we asked participants how they performed autosuggestion. In addition, it is known from hypnosis but also other forms of heterosuggestion that some participants are more suggestible, whereas others seem to be more resistant to suggestive influences^58–61^. This could be due, for example, to individual differences in absorption (i.e., the skill to ignore distractions and become more immersed in the experience)^62^ as well as in experiencing responses as nonvolitional due to reduced cognitive control abilities (i.e., dissociated control abilities)^63^. Individual differences in such factors could explain variability in our results. Another factor could be differences in the cognitive strategies used to solve the autosuggestion task, such as mental imagery or verbal reiteration^64^, but also differences in the level that these strategies can be performed (e.g., differences in vividness of auditory or visual imagery or individual differences in the strength, quality and frequency of inner speech)^65,66^. Evidence also exists that personality traits (e.g., levels of optimism) play a role in responsiveness to suggestions^67^.

It could also be argued that the use of autosuggestion in our study was aimed at modifying the stimulus characteristics rather than enhancing or decreasing tactile perception. A further experimental design could involve instructing participants to apply autosuggestion to increase or decrease the sensitivity of their finger, thus directly targeting the modulation of perceptive qualities of the finger, rather than modulating the characteristics of the stimulus itself.

Another potential confound is the small sample size (see “Methods” section, Experiment 1). To reduce the impact of using small sample size, we analysed our data using the non-parametric Wilcoxon Singed-Ranks test. In this way, we extracted the exact p-values based on actual distributions of the data. More importantly, we replicated our results of Experiments 1 and 2 with an independent participant cohort. However, given our sample size, the number of participants was too low to perform reliable correlations between PSE values and self-reported scores, such as expectancies, suggestibility, and self-efficacy reports (see^68^). Thus, further research with a larger sample size is necessary to confirm our findings and draw more robust conclusions. This is especially necessary, given the large variability observed in the actual coupling between frequency and intensity. This does not only concern the already described Békésy and reversed-Békésy effects, but also the strength of the effects themselves. Part of this variability might come from the inherent difficulty in this task of separating the amplitude and frequency components of the vibrotactile stimuli. Even though we trained participants to discriminate between the two features, and we are confident participants were able to do so, the task is still difficult, and participants could present differences in the ability to separate intensity from frequency. This might have produced an increase in participants for which data could not be properly fitted.

## Concluding remarks

In this study, we introduce a novel method for investigating the impact of autosuggestion on discriminative touch while minimising the influence of response biases and demand characteristics. The experimental design presented here could be extended in the future to explore other types of suggestion, such as placebo or heterosuggestion. We observed that the inner reiteration of a thought alters participants’ tactile perception. The mechanisms underlying these changes are, however, unknown, and the fact that we observed opposite effects for the positive versus negative association group might suggest that the effects of autosuggestion in this task reflect a change in a generalised magnitude system. Despite the acknowledged limitations, our findings offer initial empirical evidence suggesting that individuals may be capable of influencing their perception of tactile intensity through internally repeated thoughts, indicating a potential advantage of utilizing autosuggestion, both in everyday life as well as in the clinical context. Nevertheless, our research underscores the importance of conducting additional studies to systematically address response biases when investigating the effects of suggestion.

## Data Availability

All data, analysis scripts, have been made publicly available via OSF and can be accessed at https://osf.io/7hd5a/?view_only=321a7ad916e64f53a11596eccd55bd7e.
